# A Synthesized Glucocorticoid- Induced Leucine Zipper Peptide Inhibits Retinal Müller Cell Gliosis

**DOI:** 10.3389/fphar.2018.00331

**Published:** 2018-04-06

**Authors:** Ruiping Gu, Xinyi Ding, Wenyi Tang, Boya Lei, Chen Jiang, Gezhi Xu

**Affiliations:** ^1^Department of Ophthalmology, Eye and ENT Hospital of Fudan University, Shanghai, China; ^2^Shanghai Key Laboratory of Visual Impairment and Restoration, Fudan University, Shanghai, China; ^3^Key Laboratory of Myopia of State Health Ministry, Fudan University, Shanghai, China

**Keywords:** synthetic glucocorticoid-induced leucine zipper peptide, Müller cell, gliosis, NF-κB p65, LPS

## Abstract

**Purpose:** The anti-inflammatory activities of protein glucocorticoid-induced leucine zipper (GILZ) have been demonstrated *in vivo* and *in vitro*. Here, we examined the potential effect of a synthetic peptide derived from the leucine zipper motif and proline-rich region of GILZ on suppressing inflammatory responses in primary cultured rat Müller cells.

**Methods:** Peptides were selected from amino acids 98–134 of the GILZ protein (GILZ-p). Solid-phase peptide synthesis was used to generate the cell-penetrating peptide TAT, which was bound to the amino terminus of GILZ-p. Primary cultured retinal Müller cells were stimulated with lipopolysaccharide (LPS) alone or in combination with different concentrations of GILZ-p, and the interaction of GILZ-p with nuclear factor (NF)-κB p65 in Müller cells was investigated by western blotting, immunoprecipitation, and immunofluorescence. The expression of the Müller cell gliosis marker glial fibrillary acidic protein (GFAP), functional protein aquaporin (AQP)-4, and the inflammatory cytokines interleukin (IL)-1β, tumor necrosis factor (TNF) α, intercellular adhesion molecule (ICAM)-1, and monocyte chemoattractant protein (MCP)-1 was measured by Western Blotting. The concentration of those cytokines in culture medium was measured by using Enzyme-Linked Immunosorbent Assay.

**Results:** The synthesized GILZ-p, which was water-soluble, entered cells and bound with NF-κB p65, inhibiting p65 nuclear translocation. GILZ-p inhibited the LPS-induced expression of GFAP, IL-1β, TNFα, ICAM-1, and MCP-1 in Müller cells and prevented the LPS-induced downregulation of AQP4.

**Conclusions:** These results indicate that GILZ-p interacted with NF-κB p65 and suppressed p65 nuclear translocation, thereby inhibiting inflammatory cytokine release and Müller cell gliosis.

## Introduction

Sight-threatening intraocular inflammation accounts for a significant proportion of visual disability and is involved in many ocular diseases, including those of infectious, non-infectious, and multiple para-inflammatory types (Durrani et al., 2004; Deibel and Cowling, 2013; Forrester et al., 2013; Huerva et al., 2015). The mainstay of treatment for these diseases is immunosuppression, and steroids are commonly used as the first-line treatment followed by a range of second-line immunosuppressants (Jabs and Akpek, 2005; de Smet et al., 2011; Durrani et al., 2011; Nguyen et al., 2011). However, the side effects of these drugs, including a recognized mortality rate mostly related to malignancies, are numerous and debilitating, and the need for safer and more effective therapies has been highlighted (Carnahan and Goldstein, 2000; Youssef et al., 2016).

Glucocorticoid-induced leucine zipper (GILZ), which was first described as a glucocorticoid-induced protein (D’Adamio et al., 1997), belongs to the leucine zipper protein family (Ayroldi and Riccardi, 2009; Pinheiro et al., 2013). It is a dexamethasone-inducible gene that mediates glucocorticoid (GC) actions in a variety of cell types, such as in T cell, B cell, macrophage and endothelium cell. GILZ can control cell activities, such as activation and differentiation, mainly through its ability to homo-and hetero-dimerize with partner proteins, such as NF-κB, Ras, and AP-1 et al (Ayroldi et al., 2001; Delfino et al., 2006; Di Marco et al., 2007; Cheng et al., 2013; Hahn et al., 2014; Ronchetti et al., 2015). A number of *in vitro* and *in vivo* studies using animal models of inflammatory diseases demonstrate an anti-inflammatory role for GILZ (Delfino et al., 2006; Di Marco et al., 2007; Hahn et al., 2014; Ronchetti et al., 2015). Our previous studies showed that GILZ suppressed inflammatory reactions by inhibiting the nuclear translocation of NF-κB p65 (Gu et al., 2017a,b,c), a nuclear transcription factor that acts as a master regulator of inflammatory responses. Once activated, the p65 subunit translocates into the nucleus and mediates the transactivation of inflammatory genes (Grilli and Memo, 1999; Calzado et al., 2007; Menghini et al., 2016; Raish et al., 2018). The C-terminal region of GILZ contains eight prolines (P), eight glutamic acid (E) residues, and five PxxP sequences. Such sequences were previously shown to mediate protein–protein interactions (Holt and Koffer, 2001). GILZ physically binds to the amino terminal rel homology domain of p65 through an amino terminal dimerizing leucine zipper motif and a proline-rich carboxy terminus (Ayroldi et al., 2001; Di Marco et al., 2007). In the present study, we explored the effect of the synthetic peptide GILZ^98-134^ derived from the leucine zipper motif and proline-rich region of GILZ on inflammatory responses induced by lipopolysaccharide (LPS) in primary cultured rat Müller cells.

Müller cells are specialized radial glia with nuclei in the inner nuclear layer and a radial process that extends apically and basally to span the width of the retinal epithelium to provide structural and metabolic support for retinal neurons (Bringmann et al., 2006; Reichenbach and Bringmann, 2013). All pathogenic stimuli could activate Müller cells and induce Müller cells gliosis. The gliosis of Müller cells could induce oxidative stress, secrete numerous inflammatory cytokines and exert glutamate toxicity on photoreceptors and neurons (Lenkowski and Raymond, 2014). So the Müller cell was used in present study to verify the anti-inflammatory activity of GILZ-p in retina.

## Materials and Methods

### Generation of GILZ-p

The peptides shown below were synthesized and purified by solid-phase peptide synthesis using an automatic peptide synthesizer (Symphony; Protein Technologies, Tucson, AZ, United States) by ChinaPeptides Co., Ltd. (Shanghai, China). Intracellular delivery of the GILZ peptide was achieved by connecting the cell-penetrating peptide TAT (YGRKKRRQRRR) to the amino terminus of the GILZ^98-134^ peptide (YGRKKRRQRRRKTLASPEQLEKFQSRLSPEEPAPEAPETPEAPGGSAV) (GILZ-p). A 6× His tag (HHHHHH) was added to the carboxyl terminus of GILZ-p for immunoprecipitation measurement. TAT and His-tag-TAT served as control-peptides (Control-p). All peptides were of 98% purity as confirmed by high-performance liquid chromatography.

### Rat Primary Retinal Müller Cell Culture

All procedures were approved by the Animal Ethics Committee of the Eye and ENT Hospital of Fudan University, China, and were conducted in accordance with the Association for Research in Vision and Ophthalmology’s statement on the use of animals in ophthalmic and vision research.

Rat primary retinal Müller cells were cultured from 2 to 3-day-old neonatal Sprague Dawley rats. About 60 neonatal Sprague Dawley rats were used. Animals were euthanized and their eyes enucleated. After removing the anterior segment, the retina was collected, rinsed with phosphate buffered saline (PBS, Gibco, Grand Island, NY, United States), and digested with 0.25% trypsin (Gibco, 37°C, 5 min). After centrifugation (5 min, 1200 rpm), the cells were transferred to a sterile centrifuge tube, and 8–10 mL of prewarmed cell culture medium (DMEM F12 supplemented with 10% fetal bovine serum) was added to resuspend cells (DMEM F12 and fetal bovine serum were from Gibco). The resuspended cells were transferred to a T75 flask precoated with gelatin-based coating solution, which was placed in a humidified, 5% CO_2_ incubator at 37°C. The culture medium was replaced on the third day to remove non-adherent cells and replenish nutrients, and medium was replaced every 2 days thereafter. After the mixed culture reached confluence, the flasks were subjected to mechanical shaking to remove loosely attached primary microglia. The remaining cells, which were firmly attached to the bottom and consisted of 90% Müller glia, were subcultured twice to improve cell purity. The final purity of Müller glia in culture was >98%.

### Cell Viability

The viability of Müller cells was determined by the cell counting kit-8 (CCK-8) assay. Briefly, 0.5 × 10^5^ cells per well were seeded in 96-well plates and allowed to attach overnight. Then, the culture media were replaced with fresh media containing different concentrations of GILZ-p (0, 10, 50, 100, and 150 μM). After 24 h, 10 μL CCK-8 (Dojindo, Kumamoto, Japan) solution was added to each well, and the plates were incubated for another 2 h. Measurements of optical density were obtained at a wavelength of 450 nm using a spectrophotometer.

### Treatment of Müller Cells

Lipopolysaccharide was first dissolved in PBS (1 mg/mL) and then diluted in DMEM F12 culture medium to a concentration of 1000 ng/mL. The primary cultured retinal Müller cells were stimulated with 1000 ng/ml LPS or 1000 ng/ml LPS + GILZ-p (0.01, 0.1, 1, or 10 μM). At 24 h after stimulation, the Müller cells were collected and suspended in cell lysis buffer (Cell Signaling Technology, Beverly, MA, United States) containing phosphatase and protease inhibitors (Roche Diagnostics, Indianapolis, IN, United States) and stored at -80°C until further use.

### Western Blotting

Nuclear proteins and cytoplasmic proteins were separated using NE-PER Nuclear and Cytoplasmic Extraction Reagents (Thermo Scientific, Rockford, IL, United States). Equal amounts of proteins (40 μg) were loaded and separated on SDS-PAGE and transferred to polyvinylidene difluoride membranes (Millipore, Billerica, MA, United States). The membranes were blocked in 5% non-fat milk at room temperature for 1 h and incubated with the following antibodies: anti-monocyte chemoattractant protein (MCP)-1 (ab25124; Abcam, Cambridge, United Kingdom), anti-intercellular adhesion molecule (ICAM)-1 (ab171123; Proteintech, Chicago, IL, United States), anti-IL1β (ab9787; Abcam), Anti-tumor necrosis factor (TNF)α (PB0270, Boster Biological Technology, Wuhan, China), rabbit anti-p65 polyclonal antibody (ab16502; Abcam), anti-phospho-NF-κB p65 (Ser536) rabbit monoclonal antibody (3033P; Cell Signaling Technology, Beverly, MA, United States), anti-AQP4 (300-314, Alomone Labs, Jerusalem, Israel), anti-glial fibrillary acidic protein (GFAP) (ab10062, Abcam), or rabbit anti β-actin antibody (ab69512; Abcam) overnight. After washing the membranes three times, they were incubated with the appropriate secondary antibodies followed by chemiluminescent detection (Pierce Biotechnology, Rockford, IL, United States). Chemiluminescent images were captured using a Kodak Image Station 4000 MM Pro (Carestream, Rochester, NY, United States) and analyzed with Image-Pro Plus (ver. 6.0; Media Cybernetics, Bethesda, MD, United States). To detect the distribution of NF-κB p65 in Müller cells, nuclear proteins and cytoplasmic proteins were tested separately. Müller cells were treated with 10 μM GILZ-p with or without 1000 ng/ml LPS for 1 h. Cells were then collected and separated using NE-PER Nuclear and Cytoplasmic Extraction Reagents (Thermo Scientific). The band intensity was quantified and normalized against internal controls. Densitometry ratios were normalized to either total β-actin or lamin B (nuclear protein) as appropriate.

### Immunofluorescence

Müller cells were cultured on 6-well plates and starved in serum-free DMEM F12 for 24 h. The cells were then treated with 10 μM fluorescein isothiocyanate (FITC)-labeled GILZ-p for 6 and 12 h, respectively. After treatment, the Müller cells were washed with PBS, fixed in 4% paraformaldehyde (Sigma-Aldrich Corp., St. Louis, MO, United States), and permeabilized in 0.1% Triton X-100 (Promega, Madison, WI, United States) for 10 min. The cells were counterstained with 4′6-diamidino-2-phenylindole (DAPI) (Sigma-Aldrich Corp.) and examined under a laser confocal microscope (Leica Microsystems, Wetzlar, Hesse-Darmstadt, Germany).

### Immunoprecipitation (IP)

The primary cultured retinal Müller cells were stimulated with LPS (1000 ng/mL) + His-tag-GILZ-p (10 μM) or LPS (1000 ng/ml) + His-tag-Control-p (10 μM). After 24 h of stimulation, the Müller cells were collected and suspended in cell lysis buffer (Cell Signaling Technology) containing phosphatase and protease inhibitors (Roche Diagnostics), then centrifuged at 4°C for 10 min (10000 rpm). The supernatant was transferred to a precooled EP tube. Agarose protein A/G slurry (20 μl) was washed twice with precooled PBS buffer, centrifuged (3000 rpm) for 5 min, and then resuspended with precooled PBS to 50% dilution. To eliminate non-specific binding, the samples were pretreated with agarose protein A/G as follows: 500 μg of sample (500 μL) was incubated with 30 μL Agarose protein A/G at 4°C for 2 h; then, the slurry was centrifuged (4°C, 5 min, 3000 rpm) and the supernatant was collected. The binding of the p65 antibody to pretreated samples was performed as follows: 4 μg rabbit anti-p65 polyclonal antibody (ab16502; Abcam) was added to 500 μg pretreated samples (500 μL) at 4°C and incubated overnight at 4°C; then, 30 μl 50% agarose protein A/G was added and incubated for another 6 h at 4°C; the mixture was centrifuged (4°C, 5 min, 3000 rpm) and the beads were collected and washed three times with precooled PBS. Then, 30 μL sample buffer was added to the beads and boiled for 5 min. The eluting complex was subjected to SDS–PAGE separation for western blotting. The antibodies used in this procedure were rabbit anti-p65 polyclonal antibody (ab16502; Abcam) and Anti-6× His tag antibody (ab18184, Abcam).

### Flow Cytometric Analyses

Fluorescein isothiocyanate labeling of the carboxyl terminus of the peptide was performed for flow cytometry. Müller cells were cultured on 6-well plates and starved in serum-free DMEM F12 for 24 h. The cells were then treated with 10 μM FITC-labeled GILZ-p for different time (0, 5, 10, and15 min, respectively), collected and fixed using 4% paraformaldehyde in PBS for 10 min, and analyzed by flow cytometry.

### Enzyme-Linked Immunosorbent Assays

The culture supernatant concentrations of TNF-α, ICAM-1, MCP-1, and IL-1βwere measured after stimulation of Müller cells using ELISA kits (TNF alpha Rat ELISA Kit, ab46070, Abcam; Rat ICAM-1 ELISA Kit, EK0372, BOSTER; MCP-1 Rat ELISA Kit, ab100778, Abcam; and Interleukin-1 Beta Rat ELISA Kit, ab100768, Abcam; respectively). The cell culture supernatant samples were measured without dilution. The absorbance of each well was measured on a microplate reader according to the manufacturer’s instructions.

### Statistical Analysis

Statistical analyses were performed using SPSS for Windows Version 17.0 (SPSS Inc., Chicago, IL, United States). The Mann–Whitney *U*-test was used for comparison of two groups. The IC50 value of GILZ-p was calculated.

## Results

### GILZ-p Associated With Müller Cells

**FIGURE 1:**
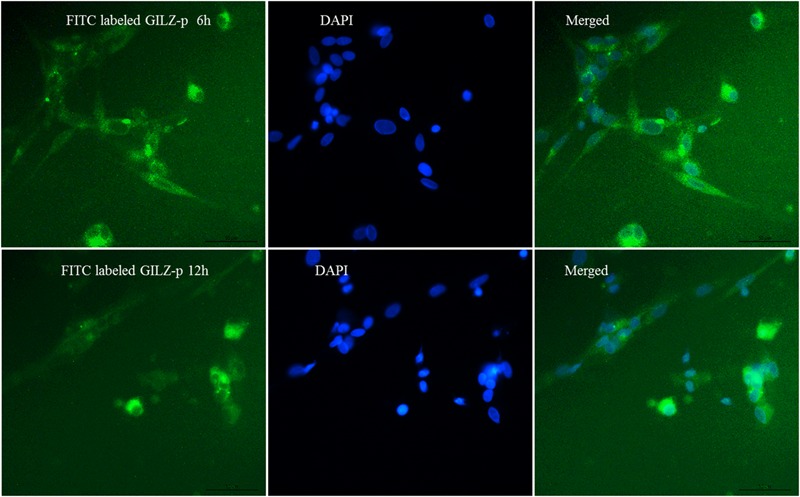
The fluorescein isothiocyanate (FITC) labeled GILZ protein (GILZ-p) (10 μM) stimulated the Müller cells for 6 and 12 h, respectively. The aggregation of GILZ-p in Müller cells was assessed by immunofluorescence. Green indicates FITC labeled GILZ-p and blue indicates 4′6-diamidino-2-phenylindole (DAPI)-stained nuclei. Scale bar: 50 μm.

The synthesized GILZ-p was water-soluble and fully dissolved in culture medium. The flow cytometric analyze showed that about 7.92% Müller cells successfully associated with GILZ-p at 15 min after treatment (**Supplementary Figure S1**). The immunofluorescence measurement also demonstrated that the GILZ-p aggregated in the cytoplasm at 6 and 12 h after treatment (**Figure 1**).

### GILZ-p Showed No Cell Toxicity

**FIGURE 2:**
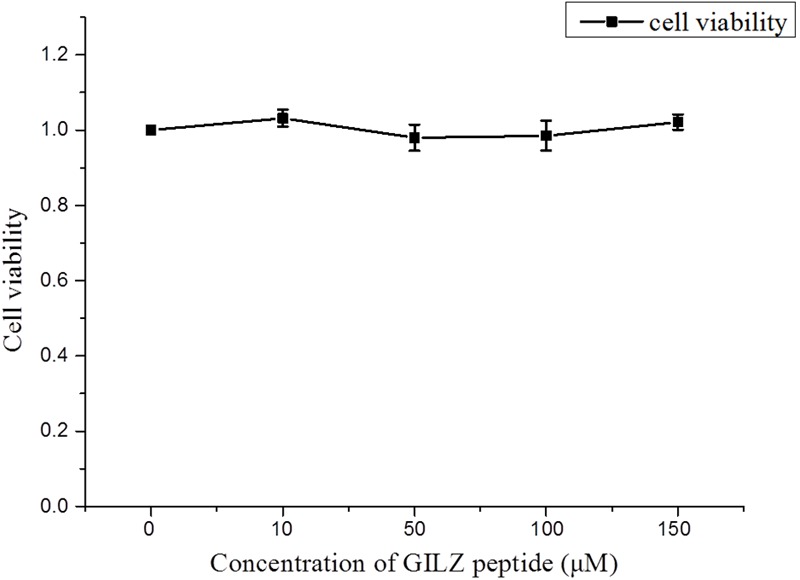
The cytotoxicity of GILZ-p was measured with the cell counting kit-8 (CCK-8) kit. At 24 h after treatment with different concentrations of GILZ-p, the viability of Müller cells was measured by the CCK-8 assay. Compared with the control group, 10, 50, 100, and 150 μM GILZ-P showed no detectable toxicity. The Mann–Whitney *U*-test was used for comparison of two groups. *n* = 6 for each group. ^∗^*P* < 0.05, ^∗∗^*P* < 0.01.

The cytotoxicity of GILZ-p was measured using a CCK-8 kit. The primary cultured retinal Müller cells were treated with different concentrations of GILZ-p (0, 10, 50, 100, and 150 μM) for 24 h. Compared with the control group, 10, 50, 100, and 150 μM GILZ-p showed no detectable toxicity (**Figure 2**).

### GILZ-p Inhibits LPS-Induced NF-κB p65 Nuclear Translocation

**FIGURE 3:**
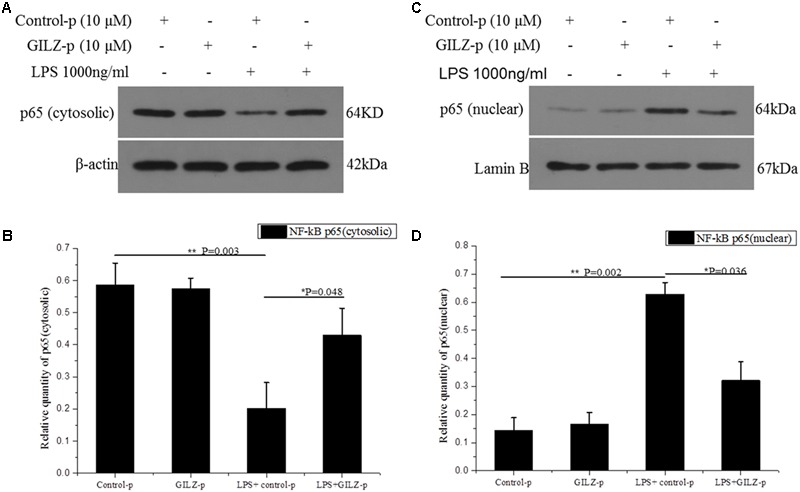
GILZ protein inhibits LPS-induced NF-κB p65 nuclear translocation in retinal Müller cells. The phosphate buffered saline (PBS) + control-peptides (Control-p), PBS + GILZ-p, lipopolysaccharide (LPS) + Control-p, and LPS + GILZ-p groups were subjected to western blot analysis of cytosolic p65 expression **(A,B)** and nuclear p65 expression **(C,D)** at 1 h after stimulation. β-actin was used as the loading control of cytosolic p65; Lamin B was used as the loading control of nuclear p65. The results of quantitative analysis, as determined by densitometric analysis, were expressed as relative to the loading control. Data represent the mean ± SE; the Mann–Whitney *U*-test was used when two groups were compared. *n* = 3 for each group. ^∗^*P* < 0.05, ^∗∗^*P* < 0.01.

Numerous studies demonstrated that GILZ exerts its anti-inflammatory effects by physically interacting with and inhibiting the activity of NF-κB (Ayroldi et al., 2001; Delfino et al., 2006; Di Marco et al., 2007; Cheng et al., 2013; Hahn et al., 2014). In previous work from our group, we used a GILZ recombinant lentivirus and showed that GILZ overexpression suppresses inflammatory reactions by inhibiting NF-κB p65 nuclear translocation (Gu et al., 2017a,b,c). In the present study, we further explored whether the synthesized GILZ-p could interact with NF-κB p65. In the normal group (PBS + Control-p or PBS + GILZ-p), p65 was mostly located in the cytoplasm, with a small amount of p65 detected in the nucleus. LPS stimulation (1000 ng/mL for 1 h) significantly increased p65 translocation from the cytoplasm to the nucleus in the LPS+ Control-p group, and this effect was suppressed by GILZ-p treatment (LPS + GILZ-p group) (**Figure 3**). This indicated that the synthesized GILZ-p suppressed LPS-induced p65 translocation.

**FIGURE 4:**
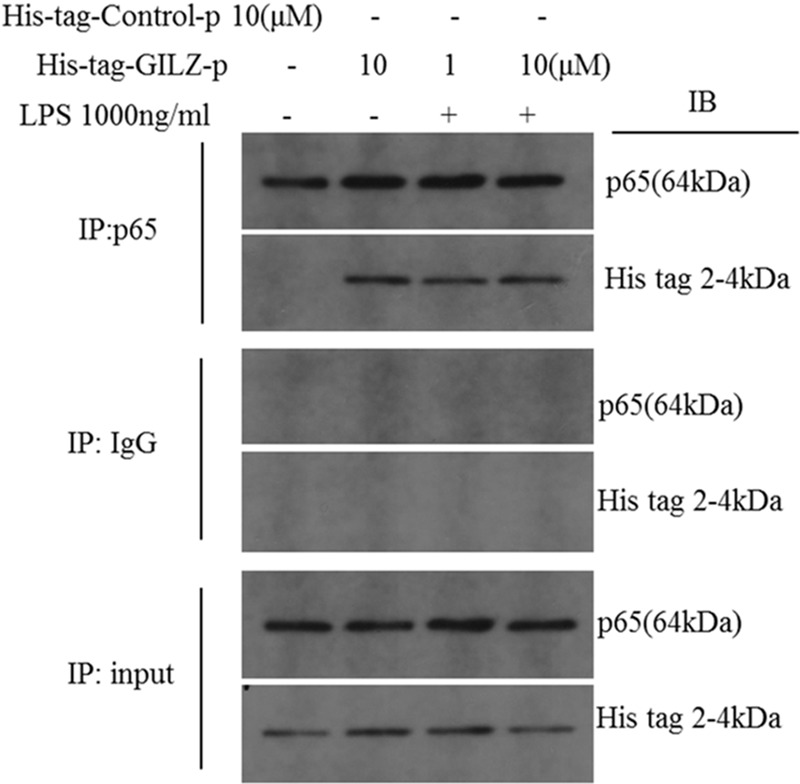
The synthesized GILZ-p interacted with NF-κB p65. Immunoprecipitation and western blot analysis showed the NF-κB p65 co-immunoprecipitation with His-tag-GILZ-p in retinal Müller cells. The IgG Co-IP served as the negative control, and the input served as the positive control.

To investigate the physical interaction between synthesized GILZ-p and NF-κB p65, the 6× His tag (HHHHHH) was added to the C terminus of GILZ-p (His-tag-GILZ-p). Co-immunoprecipitation (Co-IP) analysis in retinal Müller cells treated with His tag-GILZ-p confirmed that the synthesized GILZ-p physically interacted with NF-κB p65 (**Figure 4**).

**FIGURE 5:**
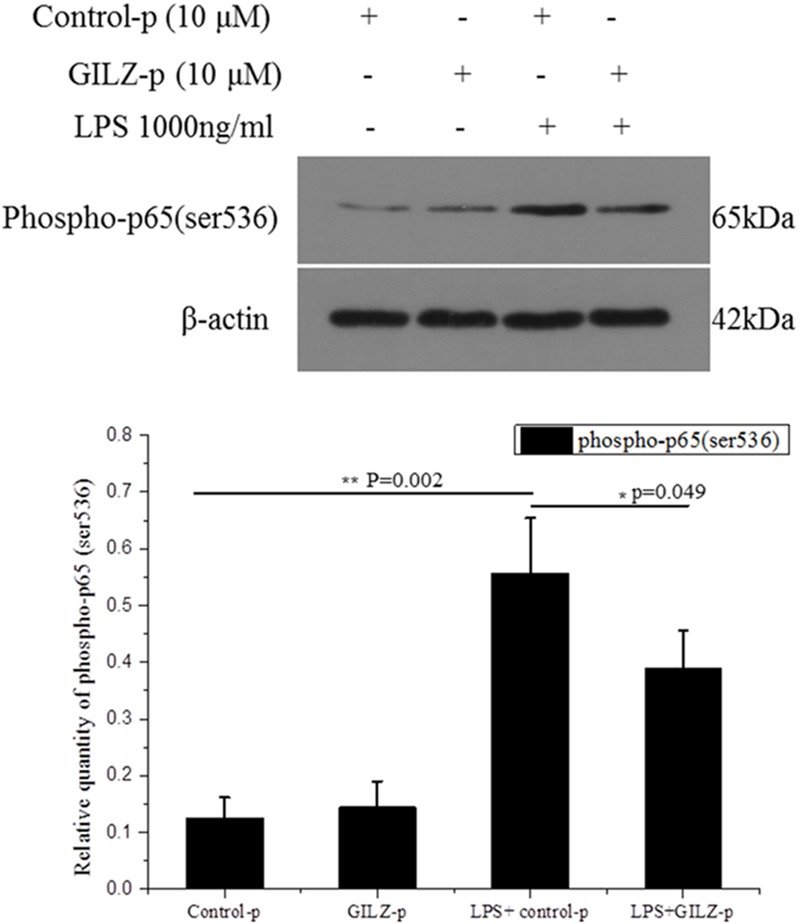
The synthesized GILZ-p inhibited LPS-induced NF-κB p65 phosphorylation in Müller cells. Western blot analysis of the phosphorylated p65 (Ser536) in the PBS + Control-p, PBS + GILZ-p, LPS + Control-p, and LPS + GILZ-p groups at 1 h after stimulation. LPS: 1000 ng/ml, GILZ-p: 10 μM; Control-p: 10 μM. β-actin was used as the loading control. The results of quantitative analysis, as determined by densitometric analysis, were expressed as relative to β-actin. Data represent the mean ± SE; the Mann–Whitney *U*-test was used for comparisons between two groups. *n* = 3 for each group. ^∗^*P* < 0.05, ^∗∗^*P* < 0.01.

In our previous study, we showed that recombinant lentivirus-mediated overexpression of GILZ promoted p65 (Ser536) dephosphorylation and inhibited NF-κB p65 nuclear translocation.(Gu et al., 2017a) In the present study, we further explored whether the synthesized GILZ peptide could promote p65 (Ser536) dephosphorylation. As shown in **Figure 5**, the level of phosphorylated-p65 at Ser536 was significantly increased at 1 h after LPS stimulation in the Control-p group. This LPS-induced increase in phosphorylated p65 was suppressed in GILZ-p treated cells. This indicated that the synthesized GILZ-p promoted p65 dephosphorylation.

### GILZ-p Protected LPS-Induced Müller Cell Gliosis

**FIGURE 6:**
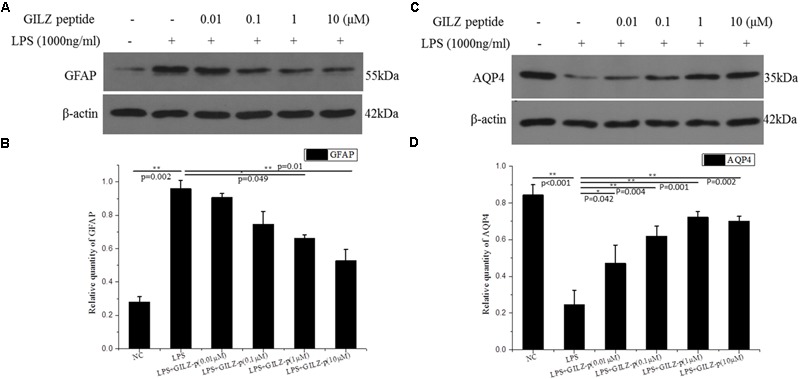
The synthesized GILZ-p inhibited LPS induced Müller cell gliosis. Western blot analysis was performed to determine the protein expression levels of glial fibrillary acidic protein (GFAP) **(A,B)**, and AQP4 **(C,D)** in Müller cells treated with 1000 ng/ml LPS in combination with different concentrations of GILZ-p (0.01, 0.1, 1, and 10 μM) for 24 h. β-actin was used as the loading control. The results of quantitative analysis, as determined by densitometric analysis, were expressed as relative to β-actin. Data represent the mean ± SE; the Mann–Whitney *U*-test was used for comparisons between two groups. *n* = 3 for each group. ^∗^*P* < 0.05, ^∗∗^*P* < 0.01.

In response to pathological stimuli, Müller cells become activated and undergo morphological, biochemical, and physiological changes in a process called Müller cell gliosis. In the present study, cultured retinal Müller cells were treated with 1000 ng/μL LPS combined with different concentrations of GILZ-p (0.01, 0.1, 1, and 10 μM) for 24 h. As shown in **Figures 6A,B**, LPS stimulation significantly upregulated the Müller cell gliosis marker-GFAP and this effect was suppressed by GILZ-p in a concentration-dependent manner. The IC50 value of GILZ-p for inhibiting the upregulation of GFAP was 11.201 μM. LPS stimulation (1000 ng/ml for 24 h) downregulated AQP4- the functional protein of Müller cells, whereas GILZ-p prevented this decrease significantly (**Figures 6C,D**). The IC50 value of GILZ-p for inhibiting the downregulation of AQP4 was 2.317 μM.

### GILZ-p Inhibited LPS-Induced Inflammatory Cytokines Secretion

**FIGURE 7:**
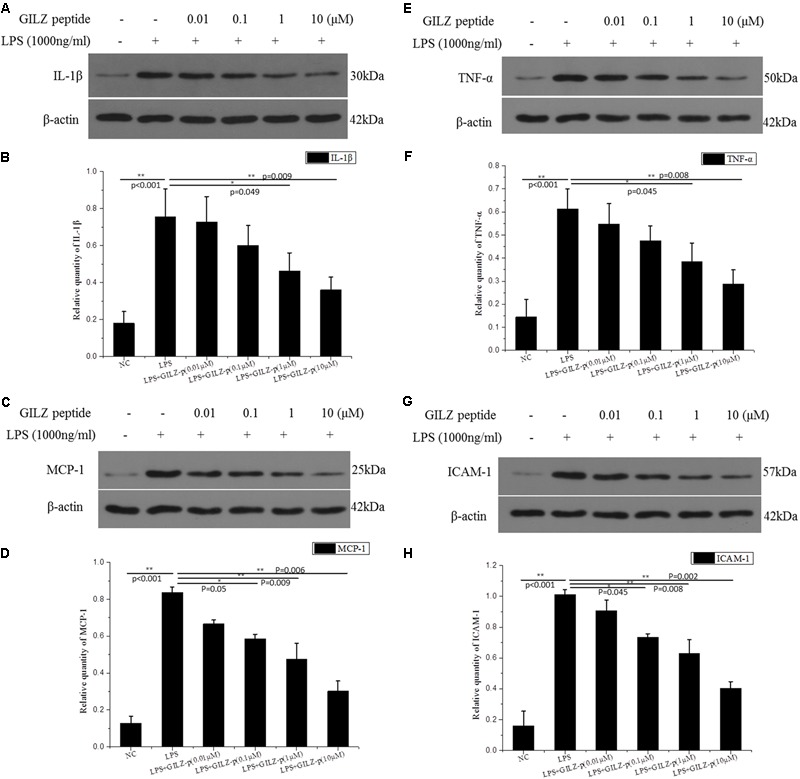
The synthesized GILZ-p inhibited LPS induced inflammatory cytokine expression in Müller cells. Western blot analysis was performed to determine the protein expression levels of pro-IL-1β **(A,B)**, MCP-1 **(C,D)**, TNF-α **(E,F)**, and ICAM-1 **(G,H)** in Müller cells treated with 1000 ng/ml LPS in combination with different concentrations of GILZ-p (0.01, 0.1, 1, and 10 μM)for 24 h. β-actin was used as the loading control. The results of quantitative analysis, as determined by densitometric analysis, were expressed as relative to β-actin. Data represent the mean ± SE; the Mann–Whitney *U*-test was used for comparisons between two groups. *n* = 3 for each group. ^∗^*P* < 0.05, ^∗∗^*P* < 0.01. TNF-α, tumor necrosis factor-alpha; ICAM-1, intercellular adhesion molecule-1; MCP-1, monocyte chemoattractant protein-1.

**FIGURE 8:**
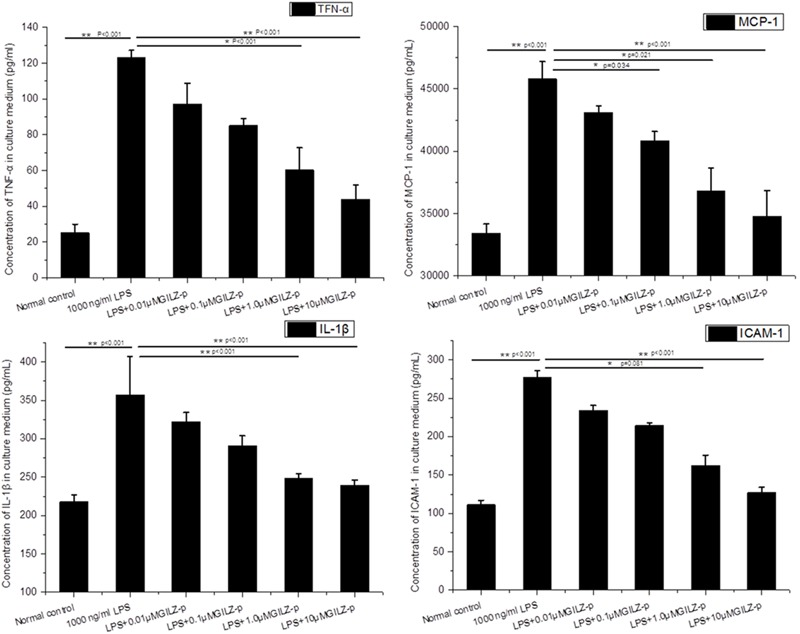
The synthesized GILZ-p decreased LPS induced inflammatory cytokine secretion in culture medium of Müller cells. The Enzyme-Linked Immunosorbent Assays (ELISA) were performed to determine the protein expression levels of IL-1β, MCP-1, TNF-α, and ICAM-1 in Müller cells treated with 1000 ng/ml LPS in combination with different concentrations of GILZ-p (0.01, 0.1, 1, and 10 μM) for 24 h. Data represent the mean ± SE; the Mann–Whitney *U*-test was used for comparisons between two groups. *n* = 6 for each group. ^∗^*P* < 0.05, ^∗∗^*P* < 0.01.

As shown in **Figure 6**, LPS stimulation (1000 ng/ml, 24 h) significantly promoted the expression of inflammatory cytokines such as pro-IL-1β, TNF-α, ICAM-1, and MCP-1 in retinal Müller cells, whereas GILZ-p downregulated the expression of these inflammatory cytokines in a concentration-dependent manner (**Figure 7**). The IC50 values of GILZ-p for inhibiting MCP-1, ICAM-1, TNFα, and IL-1β expression was 5.824, 7.129, 8.771, and 8.933 μM, respectively. The concentration of those cytokines in culture medium was measured by using the ELISA kits. The results were consistent with Western blot. As shown in **Figure 8** and **Table 1**, 1000 ng/ml LPS stimulation significantly increased the concentration of those cytokines in culture medium and GILZ-p could decreased the secretion of those cytokines in a concentration-dependent way.

**Table 1:** The concentration of IL-1β, MCP-1, TNF-α, and ICAM-1 in culture medium of Müller cells.

	TNF-α(pg/ml)	ICAM-1(pg/ml)	MCP-1(pg/ml)	IL-1β(pg/ml)
Normal control	25.17 ± 4.81	111.26 ± 5.78	33416.63 ± 764.20	217.89 ± 9.11
1000 ng/ml LPS	123.00 ± 4.12	276.80 ± 9.28	45795.90 ± 1386.73	357.12 ± 49.79
LPS+0.01 μMGILZ-p	97.08 ± 11.73	234.06 ± 6.71	43108.24 ± 752.36	322.32 ± 11.83
LPS+0.1 μMGILZ-p	85.22 ± 3.94	214.43 ± 3.40	40824.90 ± 752.36	290.72 ± 13.01
LPS+1.0 μMGILZ-p	60.26 ± 12.43	162.50 ± 13.41	36801.39 ± 1858.98	248.57 ± 6.31
LPS+10 μMGILZ-p	43.93 ± 8.00	127.14 ± 6.62	34775.54 ± 2079.00	239.08 ± 7.35

*The Enzyme-Linked Immunosorbent Assays (ELISA) were performed to determine the protein expression levels of IL-1β, MCP-1, TNF-α, and ICAM-1 in Müller cells treated with 1000 ng/ml LPS in combination with different concentrations of GILZ-p (0.01, 0.1, 1, and 10 μM) for 24 h. Data represent the mean ± SE; the Mann–Whitney U-test was used for comparisons between two groups. n = 6 for each group. TNF-α, tumor necrosis factor-alpha; ICAM-1, intercellular adhesion molecule-1; MCP-1, monocyte chemoattractant protein-1.*

## Discussion

Synthetic peptides are highly attractive as novel therapeutic agents in human diseases. The advantages of peptides include non-immunogenicity, inexpensive production, and the potential for high specificity (Saladin et al., 2009). To date, over 190 peptides have been tested in phase I/II clinical trials for the treatment of various diseases (Saladin et al., 2009). In this study, we demonstrated that the synthesized GILZ peptide interacted with NF-κB p65 and exerted anti-inflammatory effects in LPS-stimulated retinal Müller cells.

Glucocorticoid-induced leucine zipper, which was first described in 1997 as a glucocorticoid-induced protein, has been investigated in various cell types and animal models of inflammatory diseases. The C-terminal end (98–134 amino acids) of GILZ contains eight prolines (P), eight glutamic acid (E) residues, and five PxxP sequences. Such sequences were previously shown to mediate protein–protein interactions (Holt and Koffer, 2001). A number of *in vitro* and *in vivo* studies using animal models of inflammatory diseases demonstrate that GILZ can inhibit the inflammatory reaction, mainly through its interaction with NF-κB, Ras, and AP-1 et al (Ayroldi et al., 2001; Delfino et al., 2006; Di Marco et al., 2007; Cheng et al., 2013; Hahn et al., 2014; Ronchetti et al., 2015). We previously demonstrated that recombinant human lentivirus-induced GILZ overexpression inhibited LPS-induced NF-κB p65 nuclear translocation in retinal microvascular endothelial cells (Gu et al., 2017a). The anti-inflammatory activity of the full length TAT-GILZ fusion protein was evaluated in LPS-induced pleurisy (Vago et al., 2015) and in dinitrobenzene sulfonic acid-induced colitis (Cannarile et al., 2009). Those researches found that the full length of GILZ fusion protein could mimic the effects of glucocorticoids and inhibit inflammatory reaction. Ayroldi et al. (2001) and Di Marco et al. (2007) showed that the interaction of GILZ with NF-κB p65 was achieved by the GILZ C-terminal dimerizing leucine zipper motif and a proline-rich carboxy terminus (Ayroldi et al., 2001; Di Marco et al., 2007). Considering the C-terminal region of GILZ was the key domain that physically binds to the amino terminal rel homology domain of p65, so only GILZ^98-134^ sequence was chose in our present research. By using the automatic peptide synthesizer, the GILZ^98-134^ sequence was synthesized, rather than expressed in *Escherichia coli*. This method not only had high efficiency, but also avoided the contamination of endotoxin. This synthesized GILZ-p was water-soluble and fully dissolved in culture medium. Addition of 10 μM GILZ-p to the culture medium significantly inhibited LPS-induced NF-κB p65 nuclear translocation in retinal Müller cells. Immunoprecipitation (IP) measurement further demonstrated that GILZ-p interacted with NF-κB p65. These results indicated that the synthesized GILZ-p associated with NF-κB p65 and inhibited NF-κB p65 activity in a manner similar to the GILZ protein.

The phosphorylation of p65 at Ser536 can alter the kinetics of p65 and promote p65 nuclear translocation (Ren et al., 2014). GILZ overexpression induced by recombinant lentivirus transfection inhibited the LPS-induced phosphorylation of p65 at Ser536 in retinal endothelial cells (Gu et al., 2017a). In the present study, we tested the ability of synthesized GILZ-p to enhance p65 dephosphorylation. The results showed that GILZ-p significantly reduced the LPS-induced phosphorylation of Ser536. This supports the hypothesis that synthesized GILZ-p inhibits p65 nuclear translocation by promoting p65 dephosphorylation at Ser536.

Cell-penetrating peptides are used as efficient tools for delivering pharmaceuticals into cells (Xia et al., 2011; Chen et al., 2014; Zhong et al., 2015). Among them, TAT (YGRKKRRQRRR), which is derived from HIV and discovered as the first cell-penetrating peptide, is widely used to decorate bio-functional molecules and drug delivery systems with low cytotoxicity in cultured cells and animal models (Frankel and Pabo, 1988; Green and Loewenstein, 1988; Zorko and Langel, 2005; van den Berg and Dowdy, 2011; Bechara and Sagan, 2013; Duan et al., 2017). In the present study, the intracellular delivery of GILZ-P was achieved through ligation of TAT to the amino terminus of GILZ-p. As shown in **Figure 1** and **Supplementary Figure S1**, the TAT-bound GILZ-p successfully entered into the Müller cells after treatment. This was the first step leading to the interaction of GILZ-p with NF-κB p65 in the cytoplasm of cells.

In response to pathological stimuli in the retina, including inflammation, photic damage, retinal trauma, ischemia, retinal detachment, glaucoma, and diabetic retinopathy, Müller cells undergo gliosis. This phenomenon is characterized by proliferation, upregulation of the intermediate filament GFAP, activation of extracellular signal-regulated kinases 1 and 2 (ERK1/2), and alterations in the expression of functional proteins including AQP4, Kir4.1, and glutamine synthetase (Eisenfeld et al., 1984; Lieth et al., 1998; Bringmann et al., 2006; Bringmann et al., 2009). The gliosis of Müller cells is associated with a functional uncoupling from neurons, which contributes to neurodegeneration and impedes tissue repair and regular neuroregeneration. Gliotic Müller cells have direct cytotoxic effects mediated by the release of soluble proinflammatory cytokines including TNFα (Hassan et al., 2017), IL-1β (Yego et al., 2009), ICAM-1 (Wang et al., 2010), and MCP-1 (Eastlake et al., 2016). In primary cultured retinal Müller cells, LPS (1000 ng/ml) stimulation significantly upregulated the expression of GFAP, TNFα, IL-1β, ICAM-1, and MCP-1 and downregulated the functional protein AQP4. Synthesized GILZ-p inhibited the gliosis of Müller cells, as demonstrated by the downregulation of GFAP, TNFα, IL-1β, ICAM-1, and MCP-1 and the upregulation of AQP4. The anti-inflammatory effect of GILZ-p was even detected at a concentration as low as 0.01 μM. Taken together, these results indicated that GILZ-p significantly inhibited LPS-induced inflammatory responses in retinal Müller cells. However, it remains unclear whether GILZ-p inhibits Müller cell gliosis directly leading to the inhibition of the secondary release of inflammatory factors, or whether it inhibits the release of inflammatory cytokines leading to the protection of Müller cells from gliosis. Alternatively, GILZ-p may suppress both of them. In the future, we will design experiments to answer this question.

To our best knowledge, our group was the first to design and synthesize GILZ-p *in vitro*. Connection of the cell-penetrating peptide TAT to the amino terminus of GILZ-p enabled the synthesized GILZ-p to enter cells, bind with NF-κB p65, and inhibit p65 nuclear translocation, thereby inhibiting inflammatory cytokine release and Müller cell gliosis.

## Author Contributions

RG, XD, and WT: acquisition of data for the work. RG and XD: analysis of data for the work. RG, XD, and GX: interpretation of data for the work. RG, XD, WT, BL, CJ, and GX: conception or design of the work, drafting the work or revising it critically for important intellectual content, final approval of the version to be published, and agreement to be accountable for all aspects of the work in ensuring that questions related to the accuracy or integrity of any part of the work are appropriately investigated and resolved.

## Conflict of Interest Statement

The authors declare that the research was conducted in the absence of any commercial or financial relationships that could be construed as a potential conflict of interest. The reviewer GO and handling Editor declared their shared affiliation.

## Supplementary Material

FIGURE S1 Detection of fluorescein isothiocyanate (FITC)-labeled GILZ protein (GILZ-p) in Müller cells by flow cytometry. FITC labeling of the carboxyl terminus of the peptide was performed for flow cytometry. Müller cells were cultured on 6-well plates and starved in serum-free DMEM F12 for 24 h. The cells were then treated with 10 μM FITC-labeled GILZ-p for different time (0, 5, 10, and 15 min), collected and fixed using 4% paraformaldehyde in phosphate buffered saline (PBS) for 10 min, and analyzed by flow cytometry.
