# Supplementary figures and images for: A Synthesized Glucocorticoid- Induced Leucine Zipper Peptide Inhibits Retinal Müller Cell Gliosis
Source: Front Pharmacol. 2018 Apr 6;9:331. doi: 10.3389/fphar.2018.00331 (PMC5897418; doi:10.3389/fphar.2018.00331)

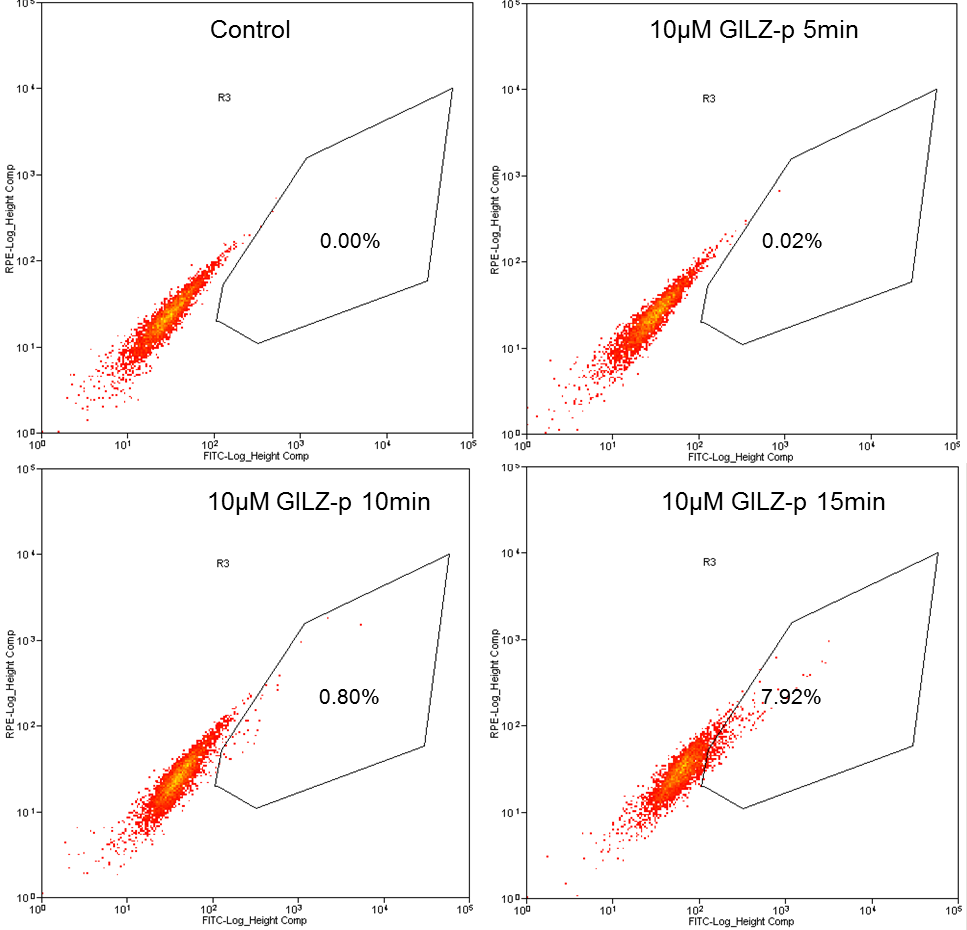

Supplement: FIGURE S1 — Detection of fluorescein isothiocyanate (FITC)-labeled GILZ protein (GILZ-p) in Müller cells by flow cytometry. FITC labeling of the carboxyl terminus of the peptide was performed for flow cytometry. Müller cells were cultured on 6-well plates and starved in serum-free DMEM F12 for 24 h. The cells were then treated with 10 μM FITC-labeled GILZ-p for different time (0, 5, 10, and 15 min), collected and fixed using 4% paraformaldehyde in phosphate buffered saline (PBS) for 10 min, and analyzed by flow cytometry. [file Image_1.TIF]
